# Synthesis of *N*-arylidene-2-(2-Phenoxyphenyl) Acetohydrazides as Anti-Inflammatory Agents

**Published:** 2011

**Authors:** Maral Shekarchi, Latifeh Navidpour, Afshin Rajabi Khorami, Mahtab shekarchi, Alireza Partoazar, Hamed Shafaroodi, Narges Rahmanipour, Abbas Shafiee, Maryam Shekarchi

**Affiliations:** a * Department of Chemistry, School of science, karaj Branch, Islamic Azad University, Karaj, Iran. *; b *Department of Medicinal Chemistry, Faculty of Pharmacy and Pharmaceutical Sciences Research Center, Tehran University of Medical Sciences, Tehran 14176, Iran. *; c *Department of Pharamcology, Faculty of Medicine, Tehran University of Medical Sciences, Tehran 14174, Iran.*; d *Department of Pharamcology, Tehran medical unit, Islamic azad University, Tehran, Iran.*; e *Department of Research and Development, Food and Drug Laboratory Research Center, Tehran, Iran. *

**Keywords:** *N*-arylidene-2-(2-phenoxyphenyl) acetohydrazide derivatives, Anti-inflammatory activity, *N*-Acylhydrazones; Non-steroidal anti-inflammatory drugs

## Abstract

Diclofenac sodium has been used for its anti-inflammatory actions for about 28 years, but since all the non-steroidal anti-inflammatory drugs (NSAIDs) suffer from the lethal gastro intestinal (GI) toxicities, diclofenac sodium is not an exception. The free –COOH group is thought to be responsible for the GI toxicity associated with all traditional NSAIDs. In the present research, the main motto was to develop new chemical entities as potential anti-inflammatory agents with no GI toxicities. A new type of 2-(2-phenoxyphenyl) acetohydrazide possessing *N*-arylidene substituents, was synthesized for evaluation as anti-inflammatory agents. The starting material 2-(2-Phenoxyphenyl) acetohydrazide was synthesized from 2-phenoxybenzoic acid in several steps according to the previous published method. Various substituted arylidene-2-phenoxynicotinic acid hydrazide derivatives were synthesized by the reaction of hydrazide 17 with selected aldehydes and screened for their potential anti-inflammatory activity. The structure of synthesized compounds was confirmed by different nuclear magnetic resonance technique, Fourier transform infrared spectroscopy (FTIR) and Mass-spectrometry data format. Qualitative structure-activity relationship data, acquired using the carrageenan-induced rat paw edema assay, showed that this group of arylidene-2-phenoxybenzoic acid hydrazides exhibit anti-inflammatory activity with significant reduction of rat paw edema (17-58% reduction in inflammation at different time intervals) in comparison with control group and a moderate to good activity range in comparison with diclofenac as the reference drug. Compounds 9a, 9d and 9e exhibited the most prominent and consistent anti-inflammatory activity. The compound, *N*-(4-Chlorobenzylidene)-2-(2-phenoxyphenyl) acetohydrazide (9d), exhibited the most *in-vivo* activity (32-58% reduction in inflammation) compared to the reference drug diclofenac (35-74% reduction in inflammation) in a carrageenan induced rat paw-edema assay.

## Introduction

Non-steroidal anti-inflammatory drugs (NSAIDs) are the most prescribed drugs in the treatment of pain and inflammation, particularly for different types of arthritis (1-3). The pharmacological activity of NSAIDs exerts their anti-inflammatory activity. The inhibition of cyclooxygenase-derived (COX) prostaglandin synthesis is also responsible for the gastro intestinal (GI), renal, and hepatic side effects observed in patients undergoing long term treatment (4-9). It was discovered that cyclooxygenase exists in two isoforms, COX-1 and COX-2, which are regulated and expressed differently. COX-1 provides cytoprotection in the gastrointestinal tract (GIT), whereas inducible COX-2 selectively mediates inflammatory signals (10-11). Since most of the currently available NSAIDs in the market show greater selectivity for COX-1 compared to COX-2, chronic use of NSAIDs, including diclofenac, may elicit appreciable GI irritation, bleeding and ulceration (12). The GI damage from NSAID is generally attributed to two factors such as local irritation by the direct contact of carboxylic acid (–COOH) moiety of NSAID with GI mucosal cells (topical effect) and decreased tissue prostaglandin production in tissues which undermines the physiological role of cytoprotective prostaglandins in maintaining GI health and homoeostasis (13). Therefore, the discovery of new safer anti-inflammatory drugs represents a challenging goal for such a research area. Synthetic approaches based upon the chemical modification of NSAIDs have been taken with the aim of improving safety profile and, in turn, the therapeutic window of these NSAIDs.

**Figure 1:**
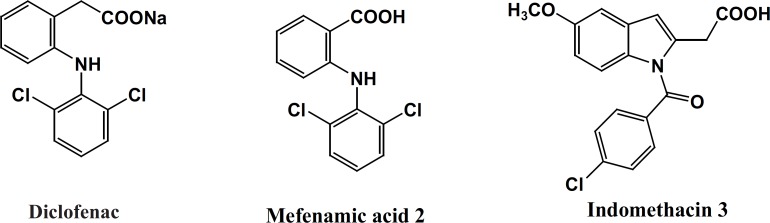
Representative examples of fenamates

Diclofenac, Mefenamic acid and Indomethacin are three traditional NSAIDs belonging to the class of fenamates (Figure 1). 

**Figure 2:**
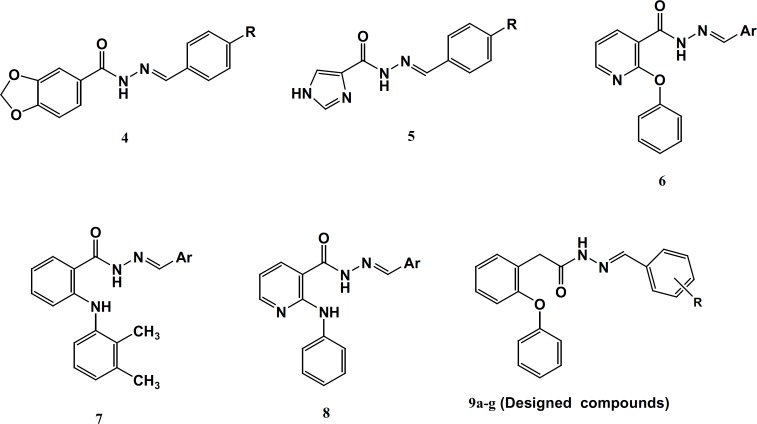
Representative examples of hydrazone derivatives 48- and designed compounds (9a-g

This class includes derivatives of *N*-phenyl (or heteroaryl) anthranilic acid that are employed for their analgesic, anti-inflammatory and anti-pyretic properties. Unlike most of the NSAIDs, the fenamates also appear to compete with prostaglandins for binding at the prostaglandin receptor site and, thus, potentially antagonizing the physiopathological effects of prostaglandins that have already been formed. Fenamates arestill endowed with most of the adverse effects induced by NSAIDs, particularly gastrointestinal (GI) bleeding, ulceration, and perforation. Several studies have described the derivatization of the carboxylate function of representative NSAIDs with amide or *N*-acylarylhydrazone having less acidic amide hydrogen resulted in an increased anti-inflammatory activity with reduced ulcerogenicity (14-17) (Figure 2). Some evidences suggest that the hydrazone moiety possesses a pharmacophoric character for the inhibition of cyclooxygenase (15). *N*-Acylhydrazones (NAH) have been widely described as potent anti-inflammatory, anti-nociceptive, and anti-platelet compounds, due to their ability to mimic the bis-allylic moiety of unsaturated fatty acids and amides, for example, arachidonic acid (AA), precursor of the eicosanoid biosynthesis, involved in the endocannabinoid system. This can be rationalized through the relative acidity in amide hydrogen of NAH group as well as its capacity of stabilizing the free radicals (14). Considering the above results and as a part of our ongoing program to design analgesic and anti-inflammatory agents (17), herein, we describe the design, synthesis and biological evaluation of a novel diverse group in *N*-acylarylhydrazone derivatives of 2-phenoxybenzoic acid 9a-g with different substituents on the terminal phenyl ring (Figure 2).

## Experimental

*Chemistry*

Melting points were determined with a Reichert-Jung hot-stage microscope (Reichert-Jung, Germany) and are uncorrected. Infrared spectra were recorded on a Nicolet Magna 550-FT spectrometer (Nicolet, Madison, WI, USA). ^1^H-NMR (500 MHz) spectra were measured through a Brucker FT-500 MHz spectrometer (Bruker Bio Sciences, USA) in CDCl_3_ or DMSO-d_6_ with tetramethylsilane (TMS) as the internal standard, where j-values (coupling constant) are estimated in Hertz. Spin multiples are given as s (singlet), d (doublet), dd (doublet of doublets), t (triplet), q (quartet), m (multiplet), and b (broad). Mass spectra were obtained, usinga Finnigan Mat triple-stage quadrupole (TSQ 70) spectrometer (Thermo-Electron, Germany). All solvents and reagents were purchased from the Fluka (Aldrich, UK), or Merck Chemical Company (Merck, Germany). Male Wistar rats used in the anti-inflammatory screens were purchased from Pasteur Institute (Karaj, Iran) and experiments were carried out using protocols approved by the ethics committee of Tehran University of Medical Sciences. Compounds 10-16 have been synthesized according to the published methods in literatures.

*2-(2-Phenoxyphenyl) acetohydrazide (17)*

A solution of compound 16 (3g, 12 mmol) and hydrazine hydrate (3g, 60 mmol) in 20 mL methanol was stirred at room temperature for 5 h. The reaction mixture poured immediately into a mixture of crushed ice water, gave a crude precipitate collected by filtration and recrystallized in ethanol/water to give 2.6 g (94%) of hydrazide derivative 17, as a light yellow amorphous solid, m.p.: 62-64^°^C;^1^H-NMR (500 MHz, CDCl_3_): δ 7.3-7.36 (m, 3H, aromatic), 7.21-7.24 (m, 1H, aromatic), 7.1 (m, 2H, aromatic), 6.94 (d, J = 7.5 Hz, 2H, aromatic), 6.87 (d, J = 7.5 Hz, 1H, aromatic), 3.76 (bs, 2H, NH_2_), 3.57 (s, 2H, CH_2_); IR (KBr): Cm^-1 ^3308 (N-H), 3037 (C-H, aromatic), 1654 (C=O), 1236 (C-O), 1096 (C-N); MS: m/z (%) 242 (M^+^, 42), 210 (68), 183 (100), 165 (32), 155 (54), 77 (45), 51 (36).

*General procedure for preparing* *N-arylidene-2-(2-phenoxyphenyl) acetohydrazides (9a-g)*

The synthesis of final hydrazones 9a-g was carried out by the reaction of hydrazide 17 and the corresponding aldehydes in ethanol. A mixture of hydrazide 17 (1.5 mmol) and the corresponding aldehyde (1.5 mmol) in ethanol (10 mL) was stirred at room temperature for 1 to 24 h in the presence of hydrochloric acid (2 drops) as the catalyst. The completion of the reaction was monitored by thin layer chromatography (TLC). The reaction mixture was concentrated under reduced pressure and was neutralized with a 10% aqueous solution of sodium bicarbonate. The resulting precipitate was filtered, washed with water, and crystallized using a suitable solvent to give 9.

*N-Benzylidene-2-(2-phenoxyphenyl) acetohydrazide (9a)*

M.p.: 169-171^°^C (Ethanol/Water); as a mixture of diastereomers in ratio of 2.59:1; ^1^H-NMR (500 MHz, CDCl_3_): δ 9.01 (bs, 0.27H, NH), 8.77 (bs, 0.28H, NH), 7.80 (s, 0.28H, =CH),7.72-7.63 (m, 1.28H, =CH, H_h_), 7.62-7.55 (m, 1.44H, H_h_), 7.49 (d, J = 7.25 Hz, 0.28H, H_d_), 7.45-7.32 (m, 4.28H, H_d_, H_f_, H_i_, X), 7.27-7.18 (m, 2.44H, H_f_, H_b_), 7.17-7.08 (m, 1.44H, H_c_, H_g_), 7.05-6.88 (m, 3.56H, H_a_, H_e_, H_c_, H_g_), 4.17 (s, 1.44H, CH_2_), 3.74 (s, 0.56H, CH_2_); IR (KBr): Cm^-1^ 3249 (N-H), 3056 (C-H, aromatic), 1677 (C=O), 1235 (C-O); MS: m/z (%) 330 (M^+^, 16), 240 (10), 227 (5), 210 (8), 183 (89), 165 (28), 155 (38), 149 (12), 134 (100), 119 (23), 104 (10), 89 (23), 77 (41), 68 (32), 55 (22).

*N-(4-Methyl-benzylidene)-2-(2-phenoxyphenyl) acetohydrazide (9b)*

M.p.: 196-198^°^C (Ethanol/Water); as a mixture of diastereomers in ratio of 2.03:1; ^1^H-NMR (500 MHz, CDCl_3_): δ 9.40 (bs, 0.68H,NH), 8.73 (m, 0.32H, NH), 7.73 (s, 0.32H, =CH), 7.64 (s, 0.68H, =CH), 7.55 (d, J = 7.25 Hz, 0.64H, H_h_), 7.49 (m, 1.68H, H_h_, H_d_), 7.36 (t, J = 7.15 Hz, 0.64H, H_f_), 7.27-7.20 (m, 2.36H, H_f_, H_b_), 7.20-7.8 (m, 3.36H, H_c_, H_g_, H_i_), 7.07-6.97 (m, 1.32H, H_a_, H_c_, H_g_), 6.97-6.87 (m, 2.32H, H_e_, H_a_), 4.17 (s, 1.36H, CH_2_), 3.72 (s, 0.64H, CH_2_), 2.38 (s, 2.04H, CH_3_), 2.36 (s, 0.96H, CH_3_); IR (KBr): Cm^-1^ 3262 (N-H), 3083 (C-H, aromatic), 1685 (C=O), 1242 (C-O); MS: m/z (%) 344 (M^+^, 5), 279 (10), 237 (3), 228 (3), 196 (3), 181 (50), 165 (20), 149 (70), 134 (65), 127 (12), 118 (20), 104 (30), 91 (32), 77 (60), 69 (62), 57 (97), 41 (100).

*N-(4-Methoxybenzylidene)-2-(2-phenoxyphenyl) acetohydrazide (9c)*

M.p.: 200-202^°^C (Ethanol/Water); as a mixture of diastereomers in ratio of 2.03:1;^ 1^H-NMR (500 MHz, CDCl_3_): δ 9.43 (bs, 0.67H, NH), 8.74 (bs, 0.33H, NH), 7.71 (s, 0.33H, =CH), 7.63 (s, 0.67H, =CH), 7.60 (d, J = 8.0 Hz, 0.66H, H_h_), 7.53 (d, J = 8.0 Hz, 1.34H, H_h_), 7.48 (d, J = 7.10 Hz, 0.33H, H_d_), 7.40 (d, J = 7.10 Hz, 0.67H, H_d_), 7.35 (t, J = 7.15 Hz, 0.66H, H_f_), 7.28-7.19 (m, 2.34H, H_f_, H_b_), 7.17-7.07 (m, 1.34H, H_c_, H_g_), 7.05-6.85 (m, 5.66H, H_a_, H_e_, H_i_, H_c_, H_g_), 4.16 (s, 1.34H, CH_2_), 3.84 (s, 2.01H, OCH_3_), 3.82 (s, 0.99H, OCH_3_), 3.72 (s, 0.66H, CH_2_); IR (KBr): Cm^-1^ 258 (N-H), 3075 (C-H, aromatic), 1677 (C=O), 1308 (C-O); MS: m/z (%) 360 (M^+^, 16), 279 (6), 257 (5), 243 (5), 218 (8), 211 (4), 194 (6) , 183 (55), 165 (21), 149 (84), 134 (100), 121 (14) , 105 (16), 97 (30), 81 (41), 69 (89), 57 (84), 43 (88).

*N-(4-Chlorobenzylidene)-2-(2-phenoxyphenyl) acetohydrazide (9d)*

M.p.: 215-218^°^C (Ethanol/Water); as a mixture of diastereomers in ratio of 2.33:1;^ 1^H-NMR (500 MHz, CDCl_3_): δ 9.34 (bs, 0.7H, NH), 8.82 (bs, 0.3H, NH), 7.79 (s, 0.3H, =CH), 7.63 (s, 0.7H, =CH), 7.59 (d, J = 8.1 Hz, 0.6H, H_h_), 7.51 (d, J = 8.1 Hz, 1.4 Hz, H_h_), 7.48 (d, J = 7.25 Hz, 0.3H, H_d_), 7.39 (d, J = 7.25 Hz, 0.7H, H_d_), 7.38-7.30 (m, 2.3H, H_f_, H_i_), 7.29-7.20 (m, 2.4H, H_f_, H_b_), 7.19-7.07 (m, 1.4H, H_c_, H_g_), 7.05-6.96 (m, 1.3H, H_a_, H_c_, H_g_), 6.95-6.85 (m, 2.3H, H_e_, H_a_), 4.15 (s, 1.4H, CH_2_), 3.73 (s, 0.6H, CH_2_); IR (KBr): Cm^-1^ 3246 (N-H), 3083 (C-H, aromatic), 1681 (C=O), 1246 (C-O); MS: m/z (%) 366 (M^+2^, 10), 330 (3), 282 (20), 257 (5),236 (12), 227 (10), 210 (8) , 196 (35), 183 (100), 195 (30), 134 (100), 165 (30) , 155 (56), 134 (89), 127 (15), 99 (7), 77 (30), 57 (28).

*N-(4-Fluorobenzylidene)-2-(2-phenoxyphenyl) acetohydrazide (9e)*

M.p.: 210-215^°^C (Ethanol/Water); as a mixture of diastereomers in ratio of 2.46:1; ^1^H-NMR (500 MHz, CDCl_3_): δ 9.24 (bs, 0.29H, NH), 8.80 (bs, 0.71H, NH), 7.80 (s, 0.28H, =CH), 7.70-7.60 (m, 1.29H, =CH, H_h_), 7.57 (dd, J = 5.5,7.5 Hz, 1.72H, H_h_), 7.48 (d, J = 7.0 Hz, 0.29H, H_d_), 7.40 (d, J = 7.0 Hz, 0.71H, H_d_), 7.36 (t, J = 7.15 Hz, 0.58H, H_f_), 7.26-7.18 (m, 2.42H, H_f_, H_b_), 7.15-7.09 (m, 1.42H, H_c_, H_g_), 7.08-6.97 (m, 3.71H, H_a_, H_c_, H_g_, H_i_), 6.96-6.85 (m, 2.29H, H_e_, H_a_), 4.16 (s, 1.42H, CH_2_), 3.73 (s, 0.58H, CH_2_); IR (KBr): Cm^-1^ 3458 (N-H), 3222 (C-H, aromatic), 1677 (C=O), 1237 (C-O), 1096 (C-N); MS: m/z (%) 348 (M^+^, 14), 256 (7), 227 (5), 210 (5), 183 (100), 165 (31), 155 (42), 149 (70), 134 (65), 134 (80), 122 (10), 107 (14), 95 (17), 77 (28), 69 (40).

*N-(2, 4-Dimethoxybenzylidene)-2-(2-phenoxyphenyl) acetohydrazide (9f)*

M.p.: 250-252^°^C (Ethanol/Water); as a mixture of diastereomers in ratio of 1.4:1; ^1^H-NMR (500 MHz, CDCl3): δ 8.00, 7.99 (bs, 1H, NH), 7.75, 7.73 (s, 1H, =CH), 7.46 (d, J = 7.5 Hz, 0.4H, H^d^), 7.39 (d, J = 7.5 H_z_, 0.6H, H_d_), 7.33 (t, J = 7.8 Hz, 0.8H, H_f_), 7.27-7.18 (m, 2.2H, H_f_, H_b_), 7.16-7.07 (m, 1.2H, H_c_, H_g_), 7.05-6.95 (m, 3.8H, H_c_, H_g_, H_a_, H_e_), 6.92-6.87 (m, 1H, H_i_), 6.60-6.52 (m, 0.4H, H_i_), 6.48 (dd, J = 2.1, 8.7 Hz, H_i_), 6.46-6.44 (d, J = 2.1 Hz, 0.4H, H_h_), 6.42 (d, J = 2.1 Hz, 0.6H, H_h_), 4.14 (s, 1.2H, CH_2_), 3.87, 3.88, 3.84, 3.83, 3.82, 3.81 (s, 6H, OCH_3_); IR (KBr): Cm^-1^ 1670 (C=O), 1207 (C-O), 1031 (C-N); MS: m/z (%) 390 (M^+^, 10), 359 (7), 227 (5), 210 (5), 183 (100), 164 (31), 150 (70), 137 (65), 122 (10), 106 (14), 95 (17),75 (28), 69 (40).

*N-(3,4,5-Trimethoxybenzylidene)-2-(2-phenoxyphenyl) acetohydrazide (9g)*

M.p.: 258-260^°^C (Ethanol/Water); as a mixture of diastereomers in ratio of 1.69:1; ^1^H-NMR (500 MHz, CDCl_3_): δ 9.55 (bs, 0.63H, NH), 8.75 (bs, 0.37H, NH), 7.76 (s, 0.37H, =CH), 7.61 (s, 0.63H, =CH), 7.47 (d, J = 7.5 Hz, 0.74H, H_d_), 7.40 (d, J = 7.5 Hz, 1.26H, H_d_), 7.36 (t, J = 7.9 Hz, 0.74H, H_f_), 7.29-7.20 (m, 2.26H, H_b_, H_f_), 7.15-7.08 (m, 1.26H, H_c_, H_g_), 7.04-6.95 (m, 1.37H, Ha, H_c_, H_g_), 6.95-6.87 (m, H_a_, H_e_), 6.88 (s, 0.74H, H_i_), 6.82 (s, 1.26H, H_i_), 4.15 (s, 1.26H, CH_2_), 3.87, 3.86 (s, 5H, OCH_3_), 3.85 (s, 4H, OCH_3_), 3.72 (s, 0.74H, CH_2_).; IR (KBr): Cm^-1^ 3243 (N-H), 3222 (C-H, aromatic), 1667 (C=O),1237 (C-O), 1065 (C-N); MS: m/z (%) 420 (M^+^, 20), 389 (10), 226 (5), 212(5), 184 (100), 170 (31), 152 (42), 149 (70), 136 (65), 121 (10), 107 (14), 90 (17), 77 (28), 69 (40).

*Anti-inflammatory activity*

The anti-inflammatory activity was determined *in-vivo* using the carrageenan-induced rat paw edema test. Edema was induced in the right hind paw of all rats by subcutaneous injection of 0.1 mL of 1% (w/v) carrageenan (Sigma-Aldrich, Dorset, UK) in saline into their footpads 0.5 h after the intra-peritoneal (IP) administration of compounds (18). The paw thickness was measured from the ventral to the dorsal surfaces using a dial caliper immediately before and then two and four h after the carrageenan injection. The edema was calculated as the thickness variation between thickness of paw before and after carrageenan injection. The anti-inflammatory activity was expressed similar to the inhibition percentage of edema when compared with the control group and was calculated using the following formula:

Inhibition% = (1 - Tt / Tc) × 100

In this formula, T_t_ and T_c_ are defined as the thickness variation of test group and control group, respectively.

*Statistics*

The results are expressed as mean ± SEM of n animals per group. The data were statistically analyzed by one-way analysis of variance (ANOVA) followed by Tukey multi comparison test.

Differences with p < 0.05 among the experimental groups were considered statistically significant.

## Results and Discussion

*Chemistry*

**Figure 3:**
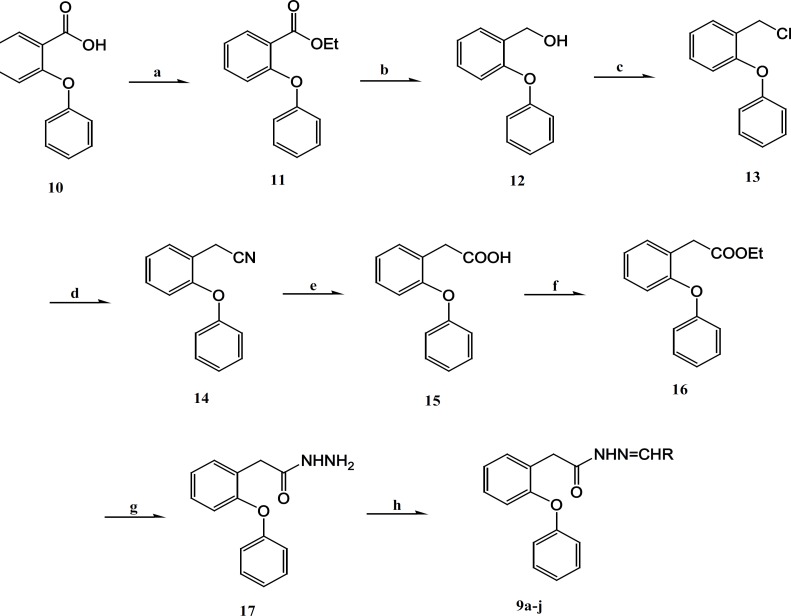
Reagents and conditions (a)thionyl chloride, abs. EtOH, reflux, 4h; (b) LiAIH4 , THF, stir, 24 h; (c) thionyl chloride, benzene/pyridine, reflux , 2h; (e) KOH , n-butanol, reflux, 2h; (f) thionyl chloride , abs. EtOH, reflux, 4h; (g) N_2_H_4_ . H_2_O. abs . MeOH, Stir, 5h; (h) aldehydes, EtOH, HCl (2 drops), stir, 1-24 h

The synthetic reactions leading to the substituted arylidene-2-(2-phenoxyphenyl)- aceto- hydrazides 9a-g are outlined in Figure 3.

2-(2-Phenoxyphenyl) acetohydrazide (17) is the key intermediate for the production of the title compounds 9a-g. Esterification of compound 10 using thionyl chloride in ethanol afforded the ethyl esters 11 in 99% yield (19, 20). Next, hydroxyl compound 12 was prepared, in 95% yield by the reduction of compound 11 with lithium aluminum hydride in Tetrahydrofuran/Tetrahydrofolic acid/Tetrahydrofolate (THF) (20). The substitution of hydroxyl to chloride compound 13 was obtained in 97% yield by the reaction of compound 12 with thionyl chloride in benzene/pyridine (21). The treatment of compound 13 with sodium cyanide and subsequent hydrolysis of the synthesized compound 14, gave 2-(2-phenoxyphenyl) acetic acid (15) in 87 and 98% respectively.Reaction of 2-(2-phenoxyphenyl) acetic acid (15) with thionyl chloride in ethanol, furnished the ethyl 2-(2-phenoxyphenyl) acetate (16) in high yield after treatment with hydrazine hydrate in absolute methanol, it resulted in the formation of the required hydrazide 17, the key intermediate of this synthesis route, in 94% yield (20, 22). Finally, the title hydrazone compounds (9a-g) were prepared in great yields by the condensation of hydrazide 17 with different substituted aromatic aldehydes in ethanol, using hydrochloric acid as catalyst (22-25). The structures of various synthesized compounds were assigned on the basis of different chromatographic and spectral studies. The physical data, Fourier transform infrared spectroscopy (FTIR), ^1^H-NMR and Mass spectral data for synthesized compounds are reported in experimental protocols.

The FTIR spectra of Schiff bases exhibited very similar features and showed the expected bands for the characteristic groups which were present in the compounds such as C–H and the C=N stretching vibrations and another specific band for Ar–C–N vibrations. Compounds of 9a–g have C=O stretching bands in the range of 1677-1685. In the proton ^1^H-NMR spectral data, all protons were seen according to the expected chemical shift and integral values. The aromatic protons appeared as multiplet peaks within the range of 6.9-7.7 ppm and the singlet signals derived from hydrazide 17 (–NH–NH_2_) structure appeared at 3.76 ppm. Methylene protons resonated as singlet at 4.1-4.2 ppm. The ^1^H-NMR spectra of compounds 9a-g displayed singlet due to –NH– groups around 8.1, 9.01 and 9.4 ppm (probably due to their ability to get exchanged with D_2_O) while each signal showed integration for one proton. For the compounds 9a-g, the signals which belonged to benzylidene group were observed at aromatic region, while the signals of –NHNH_2_ disappeared indicating the functionalization of hydrazide to hydrazone with substituted aromatic aldehydes.

The next step of this work was to determine the relative configuration of the imine double bond of arylidene benzoic acid derivatives 9a-g, in order to assure the stereoisomeric ratio essential for the complete understanding of the biological effect.The careful analysis of the ^1^H-NMR for 9a-g,enabled us to detect the presence oftwo singlet signals related to the imine hydrogen, which was attributed to respective (*E*) and (*Z*)-diastereomers. The existent ratio between the two diastereomers could be defined from the relative integration of imine-attached hydrogen in the corresponding ^1^H- NMR spectra (Table 1).

**Table1:** Physical and spectral properties of compounds 9 a-g

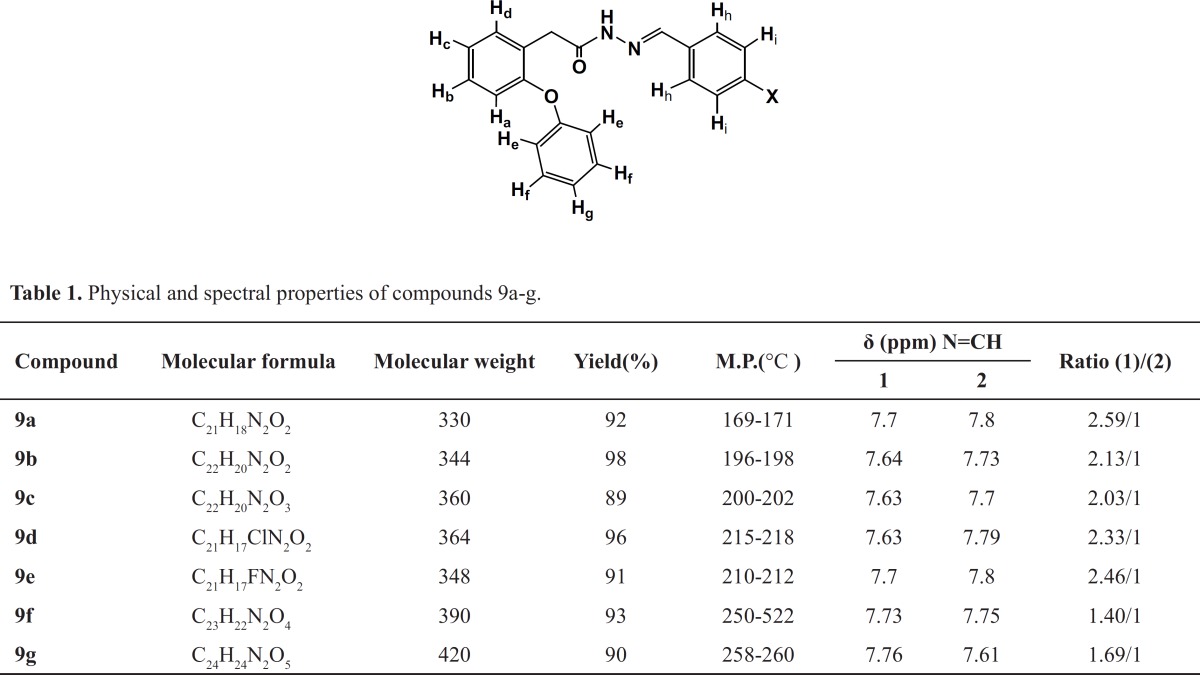

*Anti-inflammatory activity*

*In-vivo* pharmacological evaluation of 9a-g was carried out to assess their potential anti-inflammatory activity. Qualitative structure-activity relationship data acquired using the carrageenan-induced rat paw edema assay (18), showed that this group of arylidene-2-phenoxybenzoic acid hydrazides exhibit the anti-inflammatory activity with significant reduction of rat paw edema (17-58% reduction in inflammation at different time intervals) in comparison with the control group and a moderate to good activity range in comparison with diclofenac as the reference drug (Table 2). 

**Table 2:** Anti-inflammatory activities of compound 9a-g

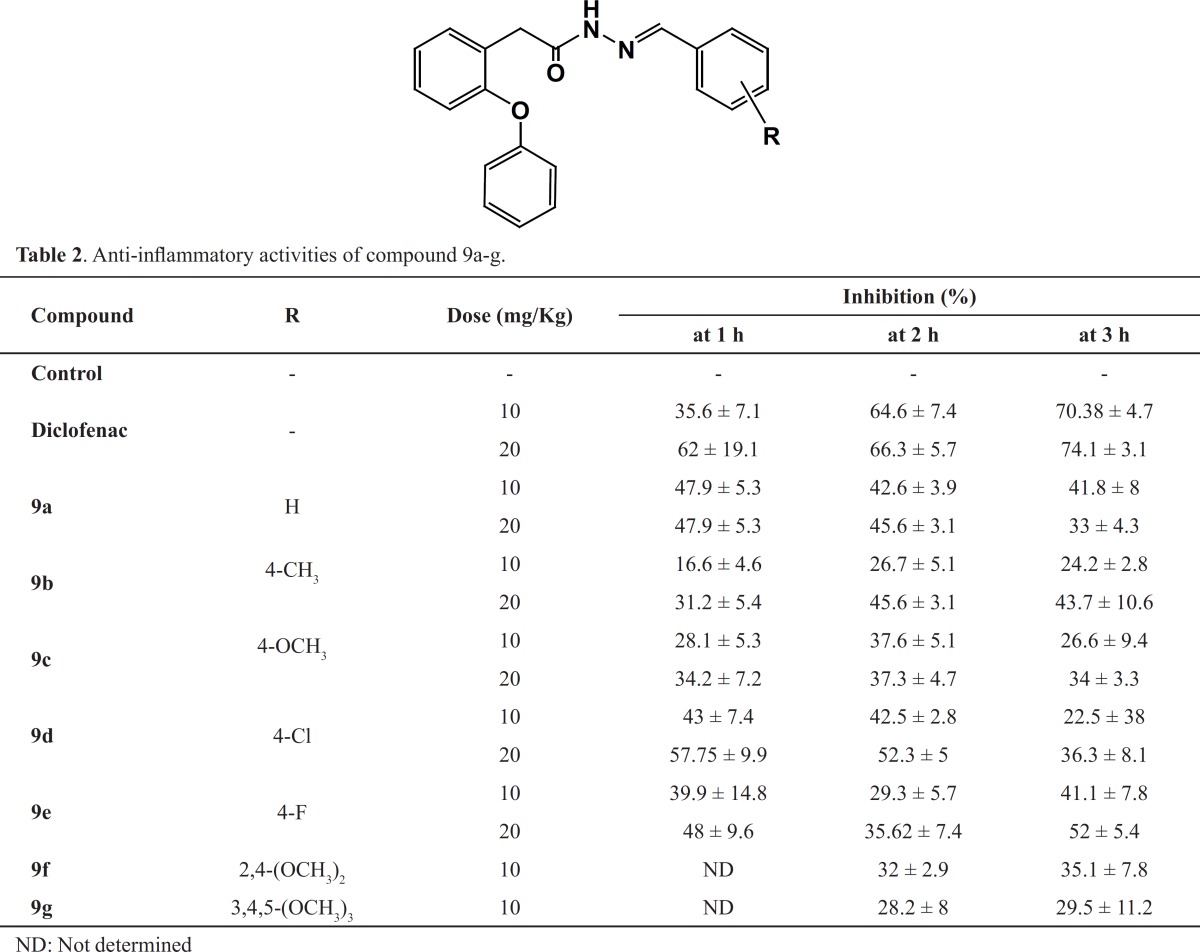

As shown in Table 2, the presence of electron-withdrawing substituents (9d-e) produced compounds with comparable activity to unsubstituted derivative (9a), while electron-donating (9b-c) substituted compounds (9b-c) showed to be less active in reduction of inflammation. Interestingly, most of the compounds showed better activity in 1 or 2 h rather than 3 h showing the faster onset of action compared to diclofenac which was possibly resulted from their more lipophilic structure. Poly-substitution in 9f-g resulted to a noticeable reduction in activity. Finally, N-(4-chlorobenzylidene)-2-(2-phenoxyphenyl) acetohydrazide (9d), was the most potent anti-inflammatory agent in this series, producing 32-58% reduction in inflammation 1 to 3 h prior to the administration of the drug.

## Conclusion

Various substituted arylidene-2-phenoxynicotinic acid hydrazide derivatives were synthesized and screened for their potential anti-inflammatory activity. Most compounds exhibited moderate to good anti-inflammatory activities in comparison with diclofenac. Compounds 9a, 9d and 9e exhibited the most prominent and consistent anti-inflammatory activity.
